# Ethical issues in genomics research on neurodevelopmental disorders: a critical interpretive review

**DOI:** 10.1186/s40246-021-00317-4

**Published:** 2021-03-12

**Authors:** S. Mezinska, L. Gallagher, M. Verbrugge, E.M. Bunnik

**Affiliations:** 1 Faculty of Medicine and Institute of Clinical and Preventive Medicine, University of Latvia, Jelgavas Str.3, Riga, LV-1004 Latvia; 2 Discipline of Psychiatry, School of Medicine, Trinity College Dublin, Dublin, Ireland; 3 Trinity Translational Medicine Institute, St. James Hospital, Dublin 8, Ireland; 4 Department of Medical Ethics, Philosophy and History of Medicine, Erasmus MC, University Medical Centre Rotterdam, PO Box 2400, Rotterdam, 3000 CA The Netherlands

**Keywords:** Research ethics, Neurodevelopmental disorders, Minors, Paediatric research, Genomics research, Critical interpretive review

## Abstract

**Background:**

Genomic research on neurodevelopmental disorders (NDDs), particularly involving minors, combines and amplifies existing research ethics issues for biomedical research. We performed a review of the literature on the ethical issues associated with genomic research involving children affected by NDDs as an aid to researchers to better anticipate and address ethical concerns.

**Results:**

Qualitative thematic analysis of the included articles revealed themes in three main areas: research design and ethics review, inclusion of research participants, and communication of research results. Ethical issues known to be associated with genomic research in general, such as privacy risks and informed consent/assent, seem especially pressing for NDD participants because of their potentially decreased cognitive abilities, increased vulnerability, and stigma associated with mental health problems. Additionally, there are informational risks: learning genetic information about NDD may have psychological and social impact, not only for the research participant but also for family members. However, there are potential benefits associated with research participation, too: by enrolling in research, the participants may access genetic testing and thus increase their chances of receiving a (genetic) diagnosis for their neurodevelopmental symptoms, prognostic or predictive information about disease progression or the risk of concurrent future disorders. Based on the results of our review, we developed an ethics checklist for genomic research involving children affected by NDDs.

**Conclusions:**

In setting up and designing genomic research efforts in NDD, researchers should partner with communities of persons with NDDs. Particular attention should be paid to preventing disproportional burdens of research participation of children with NDDs and their siblings, parents and other family members. Researchers should carefully tailor the information and informed consent procedures to avoid therapeutic and diagnostic misconception in NDD research. To better anticipate and address ethical issues in specific NDD studies, we suggest researchers to use the ethics checklist for genomic research involving children affected by NDDs presented in this paper.

**Supplementary Information:**

The online version contains supplementary material available at 10.1186/s40246-021-00317-4.

## Background

Neurodevelopmental disorders (NDDs) are an important class of mental health conditions, affecting on estimation 1 in 25 European citizens, and many others across the globe. Although as a class of conditions, it is not clearly defined, NDDs are roughly defined as conditions that arise when brain development is disturbed, leading to neuropsychiatric impairments of learning, memory, executive function, emotion and social interaction. Common NDDs include autism spectrum disorder, intellectual disability and schizophrenia. The onset of NDDs is usually during childhood or adolescence, but their effects are lasting. Despite their grave impact on patients, patients’ families and society, effective treatment options for most NDDs are lacking [1]. Because of variability in cognitive, behavioural and psychiatric symptoms of NDD, diagnosis is often difficult and time-consuming [2, 3], which hinders adequate provision of care and support for patients and their families. Also, the pathophysiological mechanisms leading to NDDs are poorly understood. Clinically NDDs frequently co-occur, and advances in genomics research also implicate shared genetic risk factors. The latter may explain co-occurrence of NDDs and offer targets for new therapies [4].

Recent research efforts are targeted at a better understanding of the pathophysiological mechanisms underlying NDDs through genomic research [5]. Genetic risk factors typically refer to common and rare genetic variation. Here the focus is on copy number variants (CNV), a form of rare variation that affects chromosomal copy, i.e. deleted or duplicated. A set of rare NDD-related CNVs (ND-CNVs) that increase risk for a range of NDDs has been identified and well-replicated [6]. The most well-known NDDs, e.g. some forms of autism spectrum disorder, intellectual disability and schizophrenia, have been associated with rare CNVs, chromosomal micro-deletions or duplications of repeated sections of DNA [5]. The most well-known NDD caused by a CNV is 22q11.2 deletion syndrome, also known as DiGeorge syndrome, that affects 1 in 4000–6000 live births [7, 8]. This deletion of 30–40 genes on chromosome 22 is responsible for a variable range of phenotypic abnormalities, including specific facial features, congenital heart and kidney problems, cleft palate, hearing loss, frequent infections and, in the majority of patients, both intellectual disability and autism spectrum disorder or attention deficit hyperactivity disorder [9]. In addition, up to 40% of 22q11.2 deletion syndrome patients develop schizophrenia or other psychotic disorders [10]. Other relatively prevalent pathogenic CNVs are located on chromosomes 3, 7, 15, 16 and 17, leading to a range of overlapping NDDs [11, 12].

As ND-CNVs are rare in the population, identification and investigation of the effects of large numbers of carrier individuals is challenging and requires coordinated large-scale international collaboration. The Maximising Impact of research in Neuro-Developmental DisorderS (MINDDS) is a trans-European research consortium established in 2017 to address this challenge. The MINDDS network aims to promote the development of research and clinical cohorts of patients with rare ND-CNVs, autism, intellectual disability and schizophrenia across Europe. MINDDS is developing standardized protocols and methodologies for phenotypic, genetic, and imaging data and sample collection, patient registration and data deposition, to enable transnational patient recruitment and data sharing, incorporating regulatory, legal and ethical requirements. Also, it facilitates the development of carrier-derived induced pluripotent stem cells for NDD drug-screening purposes.

Ethical considerations are of paramount importance for genomics research at the centre of the MINDDS network, particularly relating to early-onset brain conditions that are rare. Common considerations include involving minors in research, handling of individual research results and incidental findings, privacy and interpretation of genetic findings in a family context. Many of these issues are familiar to other types of biomedical research involving human subjects and have been extensively discussed in the bioethical literature [13]. At the same time, the activities in MINDDS raise a distinct set of ethical issues, as it involves:

1. Vulnerable children and adolescents, who, due to the impact of NDDs on cognitive functioning, are less able to provide assent [14, 15]; moreover, they may experience serious burdens associated with “deep phenotyping” (including extensive assessments of neuropsychiatric and cognitive functioning);
2. Genetic and genomic research, which has traditionally been associated with concerns related to informed consent, a right not to know, genetic discrimination, eugenics, social stigma, the return of individual research results and the handling of incidental findings [16, 17];
3. Sensitive usage of data and samples, such as linking of personal data regarding neuropsychiatric or neurodevelopment symptoms with genotype in rare conditions or the development of carrier-derived induced pluripotent stem cells for drug screening (“personalised medicine”) [18];
4. Storage, transfer and sharing of data and samples across country borders which raises questions related to data security, privacy protection, and biobank governance [19];
5. A blurring of the boundaries between clinical care and research participation which may directly contribute to finding a diagnosis [20].

Genomic research on NDDs, particularly involving minors, thus combines and amplifies existing ethical issues. Additionally, it may raise novel ethical issues, such as the communication of increased risk of psychiatric conditions in carriers of ND-CNVs for the individual and their family members and how and where counselling may be accessed. This paper presents a review of the literature on the ethical issues associated with genomic research involving children affected by NDDs as an aid to researchers to better anticipate and address ethical concerns.

## Methods

To identify ethical issues in paediatric NDD genomics research, a critical interpretive literature review was performed [21]. The description of methods and reporting of this review was done in accordance with the Preferred Reporting Items for Systematic Reviews and Meta-Analyses (PRISMA) Statement as far as applicable to critical interpretive reviews [22]. No review protocol was published beforehand.

The literature search was conducted in May 2018, in five electronic databases to identify all empirical and theoretical literature that included arguments of interest: embase.com, Medline Ovid, PsychINFO, Ovid, Web of science and Google Scholar. We systematically searched the literature for articles discussing ethical issues in NDD research. Grey literature was not sourced.

We consulted with an information specialist of the medical library of Erasmus MC (Rotterdam, The Netherlands) to develop the search strategy. Search terms used were variations on the key words: neurodevelopmental disorder, CNV, genetic, research, children and ethics. The full-search strategies for all databases are presented in the Additional file 1. The overview of the search results per database is included in the Additional file 2.

We limited the review to articles published in English in peer-reviewed journals. Furthermore, we included all articles that were published before April 30, 2018, the day the database search was conducted. Conference abstracts were excluded from the review. Quality and risk of bias assessment as is done in quantitative meta-analysis was not part of this study methodology. Publications on prenatal screening were outside the scope of this article. Case-studies were included in the first screening phase as, in these, ethical issues may have been reported. Included articles needed to address ethical concerns that might be relevant to research within the scope of MINDDS.

After retrieving records from the database search according to the inclusion/exclusion criteria, the records were entered into an EndNote library. Duplicates were removed following the method proposed by Bramer et al. [23]. As a pilot, two researchers (SM and MV) independently screened titles and abstracts of the first 100 articles identified on relevance to the research question. Similarities and discrepancies were discussed with a third researcher (EB) to improve the screening strategy. In the rest of the screening phase, SM and MV independently screened the remaining references on title and abstract. The results were compared between the two researchers. When discrepancies existed, a third researcher (EB) was involved to resolve any eligibility disagreements, and, through group discussion (SM, MV, EB), consensus was reached. For the articles included after the title and abstract screening, access to the full text of the papers was sought via various libraries; authors were contacted directly to provide their manuscripts when we were unable to access them via our institutional libraries. If an author did not reply in the set time frame of two weeks, the article was excluded on the basis on insufficient information. After the full-text screening and evaluating for eligibility, 154 publications were included in qualitative synthesis. For the detailed process of eligibility screening, see Additional file 3.

A qualitative thematic analysis was conducted on the included literature, relying on inductive reasoning. By coding sections of the full-text screened articles, specific themes and sub-themes were identified. Articles were examined, and themes compared in an iterative manner. Relevant text passages were extracted, thereby further grouping and re-grouping themes based on the content of the extracted passages.

## Results

The review included 154 articles (see Additional file 4 for the full list of the included articles). Qualitative thematic analysis of the included articles revealed themes in three main areas: research design and ethics review, inclusion of research participants, and communication of research results. The themes are presented in Table 1 and described in detail in the sections below.

**Table 1:** Themes emerging from qualitative analysis

Main themes	Sub-themes
1. Research design and ethics review	• Research design and methods • Resource allocation for research • Ethics review • Conflicts of interest
2. Inclusion of research participants	• Fair subject selection • Informed consent • Risk/benefit assessment
3. Sharing of research results	• Communication of study results • Implementation of research results • Return of individual research results • Incidental findings

### Research design and ethics review

An important sub-theme in several articles was ethical aspects of choosing *research design and methods*. Authors reiterated general principles of research ethics. For instance, researchers were warned against methodologically unsound research that exposes participants to unnecessary risks, wastes time and resources and produces misleading results [24]. Development of sound research design for NDD research starts with specification of the aim of the research which should be focused on understanding of a condition [24], as well as at its prevention, cure and amelioration [25]. Yan et al. emphasized that “researchers [..] are responsible for being aware of the requirement of validity” as a precondition to social benefit [24]. One example of ethically challenging research is the use of birth cohort studies in NDD research, where there are concerns regarding to the scope of informed consent, assent/consent as the child matures, processing sensitive information and return of results [14].

To facilitate engagement of research participants, “community-based participatory research” is proposed as a research design appropriate for NDD research. Benefits of community-based participatory research include informing researchers about questions relevant to stakeholders, better addressing stakeholders’ needs, and to “circumvent potential harms, [and] accelerate appropriate translation of biomarker discoveries” [26]. The authors specifically concluded that “re-conceptualizing biomarker discovery in autism as participatory would entail clarifying and increasing its social relevance, enhancing rather than undermining its rigor, and accelerating its intended benefits to society” [26].

Several articles highlighted ethical challenges relating to the use of specific genetic clinical and experimental techniques in research. For example, the use of whole-genome sequencing of newborns might be ethically justified only “as part of carefully developed protocols for better understanding the potential benefits and risks of this technology” [27]. Comparative genomic hybridization techniques, e.g. chromosomal microarray analysis, a method to detect CNV deletions and duplications, has “a significant likelihood of identifying a variant of uncertain significance” [27] and a potential for identifying secondary findings, posing challenges with interpretation. For example, there is an absence of conceptual clarity in relation to terms “abnormal” and “pathogenic” in research and diagnostic contexts which might lead to “unnecessary anxiety, clinical procedures, or even termination of pregnancy” [28]; therefore, use of these terms needs careful evaluation of risk/benefit ratio. Microarray analysis may yield an unprecedented volume of information and “allow inferences to be made about traits that are not necessarily associated with morbidity or mortality” [28]. Its use raises questions about incidental findings, the role of researchers and clinicians and the informed consent process.

Induced pluripotent stem cells present an opportunity to study conditions using relevant tissues which is particularly advantageous for brain-based conditions. However, the rapid implementation of induced pluripotent stem cell-based therapies leading to stem cell tourism for autism spectrum disorder has been highlighted as an ethical challenge: “parents tend to overestimate the benefits of stem cell technology while overlooking significant risks and may do so in the future with regards to emerging research involving” stem cells [29]. Finally, deep phenotyping approaches for rare NDDs frequently involve the use of neuroimaging techniques, particularly *magnetic resonance imaging* (MRI). The use of MRI raised imaging-specific issues related to data interpretation, brain-behaviour dichotomies and association versus causation [30].

The *resource allocation* for NDD research has been highlighted as a possible ethical problem. Funding allocation for rare disorder research is challenging: “[i]n general, rare diseases and neurogenetic syndromes [..] face limited availability to research, case registries, and epidemiologic surveillance” [31]. Solutions to the current lack of understanding of rare NDD were emphasized by Yan and Munir: “This includes development of research funding announcements [..] and other incentives by sponsoring agencies to accommodate inclusion of children and individuals with DD [developmental disorders], rather than resignation to their exclusion as a matter of convenience, difficulty, or cost” [32]. However, there was also concern about the overemphasis on genomics research—and the use of imaging techniques in research [29]—above non-genomics research, e.g. focused on therapies and social support. Other authors argued for transdisciplinary debate on resource allocation for different types of NDD research, as well as systematic integration of genetics into clinical research [33].

Research *ethics review* of NDD research poses challenges for research ethics committees and calls for reflection and development of specific approaches for review. There have been attempts to apply existing frameworks for ethics review to NDD research. For example, Chen et al. [34] suggested application of the seven ethical requirements for review introduced by Emanuel et al.—social or scientific value, scientific validity, fair subject selection, favourable risk-benefit ratio, independent review, informed consent, and respect for potential and enrolled research participants [13]—to NDD research. However, these requirements are general and do not guide ethics committee members how to specifically approach NDD research protocols.

Research ethics review may not always be effective. Yan et al. suggest that there can be “false negative” errors, when real problems are not detected by committees, and “false positive” errors, when ethically valid protocols are unnecessarily delayed by committees due to unnecessary concerns. These errors are “likely to be magnified for research involving children and individuals with DD compared to other categories of participants and differentially compromises research in this area” [24]. To improve the ethics review process, several authors proposed the use of community consultations [16, 35], e.g. for understanding the communities’ views on returning research results. Involvement of communities in the ethics review process is closely related to the previously mentioned participatory approaches to research design and some authors suggested “further efforts to develop participatory structures of engagement at all levels of the research and application process” [35].

Moreover, NDD research often requires international collaboration and data sharing to provide adequate sample sizes, particularly when research focuses on genotype-phenotype relationships. Since such international collaboration is essential for rare disorder research, lack of coordination of review activities between countries may lead to different requirements and a need to “interpret and satisfy the standards and procedures of two separate [research ethics committees]; this may cause significant delays in our ability to report results in a timely manner and may even result in conflicting views on whether such information should be returned to the affected family” [36]. Sharing data across country borders also leads to additional challenges for committees regarding the evaluation of safeguards for informed consent, data safety, privacy etc. [37]. This is particularly relevant to data sharing with countries that do not utilize the same safeguards as those within European Union.

Finally, some authors mentioned the possibility of *conflicts of interest* to arise, as researchers liaise with industry. This is not specific to NDD, but occurs in other types of biomedical research, as well. Alpert pointed at incentives for researchers to enrol subjects as creating potential conflicts of interest [38]. There may also be pressures in relation to commercialization of research results [39, 40]. For example, translating genomics research results into commercial DNA tests may create conflicts of interests because “[c]ompanies involved in molecular diagnostics [..] try to get [tests] approved by regulatory agencies and lobby for their use in population screening, aiming for this very high-volume market” [41].

### Inclusion of research participants

When enrolling research participants in genomics research, *fair subject selection* is an important ethical requirement [32, 34]. Because of limited cognitive abilities in some patients, NDD patients may be especially vulnerable [42, 43]. In the literature, there were concerns that burdens of research participation might accumulate on NDD patients and their family members: “choosing to recruit only family members as research participants, and particularly targeting siblings at birth, has the potential to place unfair burdens on families with a child with autism, and take advantage of their desperation” [34]. By contrast, others have argued that potentially vulnerable research participants with NDD may be disadvantaged “by the higher likelihood of exclusion from research altogether” [32]. Inclusion and exclusion criteria “based on social, racial, sexual, and other cultural biases should not be used to discriminate who will or will not enter into studies” [24]. Likewise, Fuentes and Martin-Arribas discussed the dual effects of safeguards for persons not able to provide informed consent in case of NDD research: on the one hand, these safeguards are meant to protect children, and on the other hand, they may foreclose opportunities for children to enjoy the benefits of research participation. Thus, it is feared, overprotective attitudes of research ethics committees may lead to injustice [31]. Research ethics committees must find a “golden middle” between underprotection and overprotection. In case of overprotection, “[t]he consequences of not conducting research in children and adolescents might include the perpetuation or introduction of harmful practices, failure to discover etiology of illnesses, and failure to develop new treatments for psychiatric disorders of childhood and adolescence” [44]. Overprotection of certain groups (e.g. children, psychiatric patients, ethnic minorities) may lead also to underrepresentation of these groups in research raising “the protection versus equal opportunity dilemma” [44].

Furthermore, the recruitment of participants in low- and middle-income countries raises questions regarding external researchers’ responsibilities towards research subjects and obligations to build collaborations with local researchers [45], power imbalances between researchers from wealthy countries and researchers from low- and middle-income countries, as well as regarding the impact of language and cultural differences on informed consent and communicating individual research results [36].

As a pillar of research ethics, *informed consent* was a prominent sub-theme in the papers reviewed. As most research participants with NDDs enrolled in genomic research are children or minors, consent is usually provided by parents. In relation to the informed consent process, ethical issues may arise, such as therapeutic misconception, voluntariness of participation resulting from the power imbalances in the clinician-patient relationship, or undue inducement. Research participants and their family members may see the research as a continuation of clinical care: “when genetic testing is offered, parents may not be certain the testing process is part of clinical practice of a research protocol” [46]. Blurring lines between NDD research and clinical care “may elicit a therapeutic or diagnostic misconception among participants” [47]. Parents may mistakenly believe that participation may lead to better treatments for their child, and there may be an “existential gap between what the families of paediatric research subjects understand and desire by enrolling their children in studies, and the actual uses to which data derived from their participation will be put” [48]. This aspect must be considered, e.g. “when evaluating and understanding why family members agreed to participate” [49]. When parents’ consent to participation of their child in research is given because it is the only opportunity available to them to receive a (genetic) diagnosis for their child, this may be considered undue inducement [44]. Certainly, NDD research in particular situations may lead to diagnostic and therapeutic benefits for some research subjects and their families; however, several authors highlighted that overestimation of the likelihood of personal benefit from the research is ethically unacceptable [48, 49].

Furthermore, in the context of assent and informed consent, the question how “to address children’s changing capabilities and rights as they grow and reach the age of consent” was discussed [50]. Age-gradual assent leading to informed consent and including a “continuous process of empowerment for consent paralleling the maturational process” [44] or re-consent after reaching maturity [43, 47] were suggested as possible solutions to at least some problems regarding informed consent for NDD research, especially for biobank- and biorepository-based research. At the same time, empirical data show that a majority of parents “saw themselves as ‘gatekeepers’ to their child’s medical health information up to the age of 18 and expressed a desire to learn of results without their child present” [51].

Although parents may believe that they are acting in the best interests of their child when making decisions regarding research participation, the child, when it reaches adulthood, may judge differently. It is known for some conditions that the views of the adult patient community differ significantly from the views of parents of children with the same condition. Whereas parents may see it as a disorder to be cured or treated, adults with the condition may see it not as a disorder at all, but as a way of being which ought to be respected. Perry gives an example of this problem in the context of autism: “If these two perspectives are irreconcilable, bioethicists, it seems, are presented with a great challenge in talking about consent in light of the chronicity of autism. Should the views of the adult autistic community be dismissed in favor of more traditional views about the role of parents in consenting for minors?” [52]. In particular situations, community consultation or even community consent might be useful before starting research “that may have implications for discrete population groups” [16].

Even in adult research subjects who may provide consent for research participation themselves, challenges were flagged in relation to information provision. It may, for instance, be difficult for researchers to explain the limitations of the current knowledge about genetics of NDD [45], especially to patients with incompletely developed cognitive or communicative abilities [53]. Participation in complex research projects such as MINDDS may be more difficult to understand for research participants. Therefore, the provision of appropriate materials to support their understanding and enable them to provide informed consent is required. Examples of such complex research projects might be biobank research, genetic epidemiologic research [50], epigenetic research [54], etc. As a possible solution “[v]isual aids, augmented communication systems, and ‘easy reading texts’” were mentioned [31], as well as specifically designed information sheets and informed consent forms for teenagers and their parents who in certain situations “may share the same information processing characteristics” due to heritable conditions [55]. The experience and training of researchers is deemed very important to ensure quality of informed consent and assent [25]. Involvement of a neutral clinician might improve participants’ understanding of information and help to avoid therapeutic misconception [44].

Finally, since NDD research subjects are mostly minors, it may be difficult to justify their inclusion in non-therapeutic research: “[t]he fundamental conflict is whether research can be undertaken on an individual who does not have the capacity to give or withhold consent, and which will not lead to any direct benefit for him/her, but may do so for future generations of the family and other families in a similar situation” [56]. In many countries, the criterion of minimal risk and/or minimal burdens is applied to non-therapeutic studies [34, 44], which entails that the risks and burdens of participation may be no greater than the risks and burdens incurred in daily life or in hospital life. It ties in with a more fundamental moral duty of researchers to minimize risk for research subjects [44]. Authors of several papers included in this review emphasized that *risk/benefit assessment* should not only be conducted during review by the ethics committee but should be a continuous activity throughout the research process. Most NDD research is associated with risks and burdens that may be especially salient in minors or persons with intellectual disabilities. Tabor et al. provided an example: “Parents of children with autism were concerned about the ability of research staff to manage their children’s special needs. This concern was raised with regard to the burden of the blood draw, as well as other research-related burdens for children with autism” [57]. In NDD research, risks and burdens may be minimized, for instance, by involving research personnel experienced in communication with children with limited cognitive abilities.

### Sharing of research results

Researchers are morally responsible for timely and accessible *communication of research results*, preferably through publication in open-access journals, giving other researchers the opportunity use these research results in their studies, for instance “as replication samples or for meta-analyses” [58]. Sharing of analytical methods, tools and data may advance NDD research and thus serve the interests of patients and their families [59]. For rare disorders, data sharing, especially the availability of publicly shared research databases comprised of data from patient cohorts, may help with the identification of clusters of rare cases with CNV and associated phenotype data. This could lead to the delineation of “new syndromes and furthering understanding of gene function” [60] and may help patients with timely diagnosis.

At the same time, NDD research poses specific challenges and risks to data protection and privacy. It often includes collection of large amounts of sensitive information not only about research subjects, but also about their family members, which requires protection against access by third parties [61]. Breaches of confidentiality and privacy, as well as ill-considered publishing of individual-level research results, may lead to discrimination and stigmatization of individuals of group by insurers or employers [62, 63] or by social institutions and public policies [64]. Genetic discrimination and stigmatization may affect the person’s sense of self, his or her relationships with family members and social groups, and his or her choices regarding education, employment and other life choices [65]. It may also lead to experiences of shame and guilt [66]. Genetic research on NDD may stigmatize not only persons who are affected [67], but also their relatives who are not themselves affected by a disorder: “[p]arents are also in danger of being blamed for their children’s condition when the condition is inherited” [68]. Higher risk of stigmatization may be faced in low- and middle-income countries where less support and fewer services may be available to patients and family members [25]. Also, “it is not uncommon for people to refuse participation in genetic research due to concerns about insurance or employment discrimination” [69]. Paradoxically, in some situations, confidentiality and privacy protection might be especially important within families: “Families may harbor secrets that have nothing to do with the focus of the genetic research but that participants fear will be revealed in the course of participating” [67], such as non-paternity. There is also a question whether physicians or researchers should inform family members of patients or offer assistance to research participants in doing so, when genetic tests indicate that relatives may be at risk of genetic disease [62].

Although data sharing may thus present privacy risks for research participants [40, 70], these risks can be minimized by robust data protection measures, including coding and anonymization requirements [34, 60, 71]. Also, transparency, careful planning, ethics review and informed consent form part of ethical and legal frameworks for data protection in NDD research and genetic research particularly [72].

Responsible communication of research results falls not only to researchers but also to patient and public organizations and the media. Broad communication helps to reach out to families of research participants for whom “[d]issemination of findings in an accessible format and through an appropriate source is essential” [45]. Patient organizations and journalists may help with dissemination of research results, but, like scientists, they should communicate responsibly about research [41] and recognize that it is important that language about causation is used judiciously [28]. Also tailoring NDD risk communication to different groups is seen as very important [73]. General education within society about NDD and psychiatric conditions [74], scientific literacy and adequate ethical frameworks may help to reduce risk of stigma and discrimination. Sometimes, it should be considered “whether there are some research questions that should not be asked at all” [64]. Careful choice of terminology in communication of research results is required, because terms “may be misused and cause unjust stigmatizing and blaming” [68]. An alternative perspective is that ethically sound NDD research could potentially decrease mental illness stigma [48], because an “enhanced functional, neurobiological, and molecular definition of mental illness may clarify very real scientific underpinnings of illness” [75] and may take away some of the blame, self-blame or scepticism experienced by patients with NDD or psychiatric conditions. It has also been argued that media discourses may have a positive effect on decreasing stigma and increasing public’s understanding of challenges for families of NDD patients [76].

*Implementation of research results* and translation of research findings into clinical practice raises specific ethical questions “about how to do it appropriately, ethically, and usefully” [77]. In the implementation process, researchers should be led not by “what is possible in translation from research to service, but [by] what is relevant to the individuals and families” [77]. Prenatal testing, for instance, is a controversial field of application for genomics results from NDD research, and “whether physicians should encourage such practices is a different question from whether they are legally permissible” [62]. When applied in prenatal testing, NDD research may enhance reproductive autonomy and allow for family planning but at the same time raises questions about the limited penetrance and variable expression of CNVs for NDDs, about false positive and false negative test results [25] and, therewith, for challenges for interpretation of test results and genetic counselling [78]. Premature translation and commercialization of genetic testing and neuroimaging and other biomarkers that lack strong clinical validity [69, 79] have further “raised serious ethical controversy and caused concern to many professional and regulatory bodies” [41]. One example of an ethically questionable application is the screening of potential adoptees. Although “prospective adoptive parents would have a strong interest in learning whether a child they are considering bringing into their family has a heightened vulnerability to psychiatric disorders” [62], this runs counter to existing ethical guidance for predictive genetic testing in children.

*Return of individual research results* represents a challenge in the context of genomics research. There is growing international support for a right for research participants to be offered access to their personal research data if desired [80]. Moreover, Knoppers et al. argued that researchers have a duty to return results that meet three criteria: scientific validity, clinical relevance and benefits to the participant [81]. Communication of findings meeting these criteria facilitates the individual to seek help in treating or preventing associated medical conditions [82]. Still, many researchers are reluctant to return test results because of budgetary constraints and lack of practical recommendations in existing guidelines [80]. Positive research results need to be validated, and in case of clinically relevant findings, research participants need to be referred to clinical services for follow-up. This affects not only the research budget but also clinical service provision, while it is often not clear whether follow-up benefits the research participant. Potential harms include increased stress or anxiety, lifestyle change, reducing future perspective, risk of stigmatization, discrimination and negative impact on family relationships [80]. In addition, knowing test results could increase economic risk because “it could prevent the subject from getting medical (and possibly life) insurance in the future” [44]. On the other hand, research participants are generally interested in obtaining individual research results, including genetic findings, and feedback of those results can serve as a recognition of reciprocity and help increase willingness to participate in research [80]. Other benefits of disclosing individual results, in the context where research results are validated and the person has the opportunity to access the appropriate clinical care, include reduction of uncertainty and opportunity to plan for the future. Furthermore, knowing the risk of “transmission” may help with reproductive decision-making [80]. On the other hand, families may experience emotional and psychological distress, including feelings of guilt, upon learning they passed along genetic material that caused the NDD [82]. It is important to note that psychiatric risks associated with genetic variants are often of limited or unclear clinical significance, thus rarely meeting the criteria posed by Knoppers et al. [81] and are therefore rarely reported in research contexts [62].

Finally, *incidental findings*—findings that are beyond the scope of the research question—are unavoidable for genome-wide tests and pose ethical dilemmas for researchers [83]. There are concerns that knowledge gained in the course of research may be unexpected or unwanted, especially for healthy controls [44]. On the other hand, it is stressed that incidental findings may have clinical utility for subjects or their family members [27]. Researchers are recommended to discuss preferences of children and their parents with regard to the return of incidental findings during the informed consent process [44].

## Discussion

Current research efforts in genomics of neurodevelopmental disorders raise specific ethical issues in three phases of research: research planning and ethics review, inclusion of (young) research participants and communication of research results. Ethical issues known to be associated with genomic research in general, such as privacy risks and informed consent/assent, seem especially pressing for NDD participants because of potentially decreased cognitive abilities, increased vulnerability, and stigma associated with mental health problems. As Lázaro-Muñoz and Lenk point out, “many of the ‘phenotypes’ targeted by eugenics movements, including euthanasia and sterilization programs” were psychiatric and neurodevelopmental disorders [84].

As most genomics research on NDDs is non-therapeutic and involves the inclusion of children with cognitive, behavioural and/or psychiatric disorders, it must meet high ethical standards. The risks of research participation should be no more than minimal (as compared to daily life and medical care). In genomics research, research procedures are usually minimally invasive, and when experienced and well-trained clinicians or research nurses are involved in, e.g. blood draw, the physical burden of research participation is not likely to be excessive. However, there are *informational* risks associated with participation in genomics research on NDD: learning genetic information about NDD may have psychological and social impact, not only for the patient but also for the family members. Also, there are risks related to discrimination based on genetic information by third parties. However, there are potential informational *benefits* associated with research participation, too. Notably, by enrolling in research, the participants may access genetic testing and thus increase their chances of receiving a (genetic) diagnosis for their neurodevelopmental symptoms. The genome may, potentially reveal prognostic or *predictive* information about disease progression or the risk of concurrent future disorders. One of the best-studied examples is the risk of psychiatric morbidity, notably psychosis, in patients with 22q11.2 deletion syndrome [10, 85]. It could be valuable for patients and their caregivers to learn this information.

Although the association between 22q11.2 deletion syndrome and psychiatric morbidity is strong and conveys relatively large risks, this is not typical for predictive genomic testing in NDDs. Often, it is unclear whether predictive genomic test results for psychiatric disorders associated with NDDs are clinically valid: the presence of one or more risk alleles is likely to lead to only a slightly or modestly increased absolute risk of developing the disorder. The result may not be clinically actionable, either, as the disorder itself—if it develops—will be treatable to a considerable extent, regardless of the presence or absence of a possible genetic explanation. Moreover, preventive measures are likely not to be available before symptoms arise. Therefore, knowing about a potential future increased risk does not lead to any change in health or risk management. If the benefits of a genomic test (for psychiatric co-morbidity) do not outweigh the risks, it should not be offered in a clinical, diagnostic setting. In spite of the growing international trend towards more active sharing of individual research results with participants [80], there are no obligations on the part of researchers to return research results of limited clinical validity and utility. The same applies to re-contacting patients and their families when more advanced diagnostic techniques become available, revealing additional risk information [86]: when genetic test results are of limited predictive value and/or will not affect clinical management, they need not be reported.

Given its—at times—unclear clinical validity and utility, genomic risk information on NDDs is easily misinterpreted. This is exacerbated by the blurring lines between research and clinical care. In many genomic studies of NDDs, treating physicians will enrol patients with a dual aim: to contribute to the generalizable knowledge on the genomic aetiology of NDDs and to increase the chance of finding a genomic diagnosis to the benefit of the patient and their family members. The presumed “existential gap” [48] between clinical care and research may thus not exist, and the “diagnostic misconception” may not be a misconception at all. These tensions are present in parallel ethical discussions on so-called “learning healthcare systems” [87, 88], healthcare systems in which patient samples and data are routinely collected, used for the purposes of clinical care, re-used in ongoing research activities within the hospital, possibly shared with other institutions and used again in collaborative research efforts. Any research results that may be of relevance either to the individual patient from whom the samples and/or data originate or to the community of patients will be fed back to treating physicians and can be used to improve or refine clinical care. Here, too, engagement with affected individuals and their families is important to understand what would for them be most relevant research results to be utilized.

When children—who lack the capacity to consent—are enrolled in research, parents are asked to provide proxy informed consent. At the same time, researchers are required to involve the child as much as possible in the decision-making process, provide tailored information about the research and ask for age-gradual assent. This had best be accomplished by involving research personnel experienced in communication with children with limited cognitive abilities. On the other hand, some parents may wish to protect the child against genetic information that could have adverse implications for the child. As gatekeepers [51], parents should be free to withhold information in their assessment of the best interests of the child as long as the child lacks the capacity to consent. However, there are concerns related to representation, and more specifically, to a potential disconnect between the child’s future preferences and the parents’ perceptions of the child’s (future) best interests. As said, in the autism spectrum disorder community, for instance, it is claimed that cognitively normally functioning adult patients may be better representatives of the interests of autistic children than are their parents. Parents may want the child to be treated, whereas adult patients may prefer societal interventions (e.g. public awareness, societal acceptance) over medical interventions, to improve the lives of those living with these “disorders” [89]. Gradual assent and re-consent models, which seek to involve the child as much as possible in treatment and research decision-making, may not suffice in respecting the child’s anticipated rights to autonomy, as treatments initiated in early childhood may irreversibly affect the developing child’s future preferences. These concerns merit further normative reflection.

Based on this review, we second recent recommendations for researchers who are working in genomics research activities in relation to NDDs to engage patient communities in research design and communication of research results [90]. Successful implementation of *patient*-centered approaches has so far lagged behind, but is in the interests of both patients and researchers. Furthermore, researchers should ensure adequate data protection, for the privacy risks associated with genomic data on NDDs may be especially great. When sharing data internationally, the level of data protection should be upheld to the highest standards. Also, researchers should minimize the risks and burdens associated with research participation, for instance by involving clinicians or study personnel that is especially trained to communicate with persons with reduced cognitive capacities. For dissemination purposes, research results should be presented in accessible formats and stigmatizing language should be avoided.

Moreover, we urge research ethics review committees to critically evaluate the balance of risks and benefits of genomics research participation for children with NDDs. Also, committees should assess the informed consent process and help to avoid diagnostic and/or therapeutic misconception as well as conflicts of interest. This review shows that there are concerns regarding fair subject selection: children or families with rare genomic conditions may be asked repeatedly to participate in research, and the burdens of research participation may thus fall disproportionally on these children and their siblings, parents and other family members. In most countries, systems for research ethics review are designed to evaluate *individual research projects* and not to monitor repeat participation of subjects. This issue, too, merits further scrutiny.

A limitation of this study is the complexity of the literature search. Most of the papers included in this review covered only some, but not all aspects of the type of research we meant to find ethics guidance for. An article by Rahimzadeh et al. presents an ethical framework for data sharing in paediatric genomic research [91]. A special issue of the *American Journal of Medical Genetics* Part B: Neuropsychiatric Genetics presents articles on ethical issues in psychiatric genomics research [84]. There is not yet any literature on precisely the combination of the aspects (genetics and genomics, paediatric study population, neurodevelopmental and psychiatric disorders, international setting) that characterizes the research activities of MINDDS and similar research efforts. This is what this review seeks to accomplish.

## Conclusions

Researchers should partner with communities of persons with NDDs and collaborate closely to understand the relevance of the research to the community, to determine participant-relevant research outcomes, to refine research design, to reduce burdens and risks of research participation and to work on communication and information materials in accordance with participants’ level of understanding and informational preferences. Particular attention should be paid to preventing disproportional burdens of research participation of children with NDDs and their siblings, parents and other family members.

Researchers should carefully tailor the information and informed consent procedures to avoid therapeutic and diagnostic misconception in NDD research. It is important to involve research personnel experienced in communication with children with limited cognitive abilities and their parents, to manage risks and improve communication during the research. Researchers should address children’s changing capabilities and rights as they grow and reach the age of consent in the context of NDD research.

To better anticipate and address ethical issues in specific NDD studies, we suggest researchers to use Ethics checklist for genomic research involving children affected by NDDs prepared as a result of this review (see Table 2).

**Table 2:** Ethics checklist for genomic research involving children affected by NDDs

**Research design and ethics review**	
☐ Have you considered risks and benefits for the participants (e.g. data safety, privacy, possible stigmatization or discrimination based on the research results)?	
☐ Have you considered using a community-based participatory design for (part of) your research?	
☐ Have you reflected on the use of terms “abnormal”, “pathogenic” etc. in the context of your research protocol?	
☐ Do you have access to personnel trained in handling children/persons with limited cognitive abilities?	
**Inclusion of research participants**	
☐ Have you considered accumulation of burdens for research participants and their families that might be caused by participation in numerous research studies?	
☐ How you will avoid diagnostic/therapeutic misconception in the process of informed consent?	
☐ How you will explain the difference between NDD research and clinical care to potential research participants?	
☐ Have you included in the informed consent form information on the return of individual genomic research results and incidental findings?	
☐ How you will address children’s changing capabilities and rights as they grow and reach the age of consent? Do you plan to apply age-gradual assent? Do you plan to seek re-consent after research participants reach maturity?	
☐ Have you considered that parents or research participants in certain situations may share limited cognitive abilities due to a heritable condition, and that, therefore, there might be need for specifically designed informed consent forms?	
☐ What kind of materials do you plan to use to support research participants’ and their families’ understanding about research in the process of informed consent (e.g. visual aids, augmented communication systems, easy reading texts)?	
**Sharing of research results**	
☐ Do you plan to use community consultations (e.g. with patient organizations) for understanding the communities’ views on communication of research results?	
☐ Will you develop a dissemination plan tailored to relevant patient communities?	
☐ How will you protect identities of those with rare conditions in the context of open-access publishing?	
☐ How can the research project contribute to general education within society about NDD and psychiatric conditions to reduce risk of stigma and discrimination?	

## Supplementary Information

**Additional file 1.** This file includes full-search strategy per database. Full-search strategy per database.

**Additional file 2.** The file includes overview of the search results per database. Overview of the search results.

**Additional file 3.** This file presents a flow diagram of screening process for identified articles. Flow diagram of screening process.

**Additional file 4.** The file includes list of publications included in the analysis. List of publications.

## Data Availability

Data sharing is not applicable to this article as no datasets were generated or analyzed during the current study.

## Abbreviations

CNV: Copy number variants
DD: Developmental disorders
MINDDS: Maximising Impact of research in Neuro-Developmental DisorderS
MRI: Magnetic resonance imaging
NDD: Neurodevelopmental disorders
ND-CNVs: NDD-related CNVs
PRISMA: Preferred Reporting Items for Systematic Reviews and Meta-Analyses
