# Supplementary material for: Ethical issues in genomics research on neurodevelopmental disorders: a critical interpretive review
Source: Hum Genomics. 2021 Mar 12;15:16. doi: 10.1186/s40246-021-00317-4 (PMC7953558; doi:10.1186/s40246-021-00317-4)
Supplement: Supplementary file 1 — Additional file 1. This file includes full-search strategy per database. Full-search strategy per database. [file 40246_2021_317_MOESM1_ESM.pdf]

Additional file 1: full search strategy per database

embase.com 675

('mental disease'/de OR 'mental patient'/de OR 'developmental disorder'/de OR Autism/exp OR 'schizophrenia spectrum disorder'/exp OR 'intellectual impairment'/de OR 'mental deficiency'/de OR 'psychiatry'/de OR (autis* OR schizophren* OR ((mental* OR developmental* OR intellect* OR cognit* OR psychomotor*) NEAR/3 (disease* OR disorder* OR deficien* OR disab* OR impair* OR retard* OR handicap*)) OR psychiatr*):ab,ti) AND ('ethics'/de OR bioethics/de OR 'medical ethics'/exp OR 'research ethics'/de OR 'data sharing'/de OR 'information dissemination'/de OR 'incidental finding'/de OR 'stigma'/de OR 'privacy'/de OR (ethic* OR stigma OR stigmatiz* OR discrimination* OR bioethic* OR disclos* OR (data NEAR/3 (shar*)) OR ((incidental OR unsolicit* OR secondar* OR ancillary) NEAR/3 finding*) OR privacy OR recontact OR re-contact OR consent OR (return NEAR/3 (result* OR finding*)) OR (duty NEAR/3 warn) OR (information* NEAR/3 disseminat*)):ab,ti) AND (child/exp OR adolescent/exp OR adolescence/exp OR pediatrics/exp OR childhood/exp OR 'child development'/de OR 'child growth'/de OR 'child health'/de OR 'child health care'/exp OR 'child care'/exp OR 'childhood disease'/exp OR 'pediatric ward'/de OR 'pediatric hospital'/de OR (adolescen* OR infan* OR newborn* OR (new NEXT/1 born*) OR baby OR babies OR neonat* OR child* OR kid OR kids OR toddler* OR teen* OR boy* OR girl* OR minors OR underag* OR (under NEXT/1 (age* OR aging)) OR juvenil* OR youth* OR kindergar* OR puber* OR pubescen* OR prepubescen* OR prepubert* OR pediatric* OR paediatric* OR school* OR preschool* OR highschool*):ab,ti) AND ('genetics'/de OR 'developmental genetics'/de OR 'behavior genetics'/de OR 'human genetics'/exp OR 'molecular genetics'/exp OR 'copy number variation'/exp OR 'genomics'/de OR genome/de OR 'whole genome sequencing'/de OR 'whole exome sequencing'/de OR 'functional genomics'/de OR 'induced pluripotent stem cell'/de OR 'genetic counseling'/de OR 'gene deletion'/de OR 'gene duplication'/de OR 'biobank'/exp OR 'biological sample'/de OR 'genetic analysis'/de OR 'gene sequence'/de OR exome/de OR 'genetic risk'/de OR biomaterial/de OR (genetic* OR genomic* OR genome* OR exome OR sequenc* OR (copy NEAR/3 number* NEAR/3 (variation* OR variant*)) OR (induc* NEAR/3 pluripotent* NEAR/3 (stem-cell* OR stemcell*)) OR deletion OR duplication OR biobank* OR bio-bank* OR 'biological sample*' OR biomaterial):ab,ti) NOT ([Conference Abstract]/lim) AND [english]/lim

Medline Ovid 691

(Mental Disorders/ OR Mentally Ill Persons/ OR Developmental Disabilities/ OR exp Autistic Disorder/ OR Schizophrenia/ OR Intellectual Disability/ OR Psychiatry/ OR (autis* OR schizophren* OR ((mental* OR developmental* OR intellect* OR cognit* OR psychomotor*) ADJ3 (disease* OR disorder* OR deficien* OR disab* OR impair* OR retard* OR handicap*)) OR psychiatr*).ab,ti.) AND (Ethics/ OR Ethics.fs. OR Bioethics/ OR Ethics, Medical/ OR Ethics, Research/ OR Information Dissemination/ OR Incidental Findings/ OR Social Stigma/ OR Privacy/ OR (ethic* OR stigma OR stigmatiz* OR discrimination* OR bioethic* OR disclos* OR (data ADJ3 (shar*)) OR ((incidental OR unsolicit* OR secondar* OR ancillary) ADJ3 finding*) OR privacy OR recontact OR re-contact OR consent OR (return ADJ3 (result* OR finding*)) OR (duty ADJ3 warn) OR (information* ADJ3 disseminat*)).ab,ti.) AND (exp Child/ OR exp Infant/ OR exp Adolescent/ OR exp "Child Behavior"/ OR exp "Parent Child Relations"/ OR exp "Pediatrics"/ OR "Child Nutrition Sciences"/ OR "Infant nutritional physiological phenomena"/ OR exp "Child Welfare"/ OR "Child Development"/ OR exp "Child Health Services"/ OR exp "Child Care"/ OR "Child Rearing"/ OR exp "Child development Disorders, Pervasive"/ OR "Child Psychiatry"/ OR "Child Psychology"/ OR "Hospitals, Pediatric"/ OR exp "Intensive Care Units, Pediatric"/ OR (adolescen* OR infan* OR newborn* OR (new ADJ born*) OR baby OR babies OR neonat* OR child* OR kid OR kids OR toddler* OR teen* OR boy* OR girl* OR minors OR underag* OR (under ADJ1 (age* OR aging)) OR juvenil* OR youth* OR kindergar* OR puber* OR pubescen* OR prepubescen* OR prepubert* OR pediatric* OR paediatric* OR school* OR preschool* OR highschool*).ab,ti.) AND (Genetics/ OR Genetics.fs. OR Human Genetics/ OR Molecular Biology/ OR DNA Copy Number Variations/ OR Genomics/ OR Genome/ OR Whole Genome Sequencing/ OR Whole Exome Sequencing/ OR Induced Pluripotent Stem Cells/ OR Genetic Counseling/ OR Gene Deletion/ OR Gene Duplication/ OR Genetic Testing/ OR Exome/ OR (genetic* OR genomic* OR genome* OR exome OR sequenc* OR (copy ADJ3 number* ADJ3 (variation* OR variant*)) OR (induc* ADJ3 pluripotent* ADJ3 (stem-cell* OR stemcell*)) OR deletion OR duplication OR biobank* OR bio-bank* OR biological sample* OR biomaterial).ab,ti.) AND english.la.

PsycINFO Ovid 338

(Mental Disorders/ OR Developmental Disabilities/ OR exp Autism Spectrum Disorders/ OR SCHIZOPHRENIA/ OR Psychiatry/ OR (autis* OR schizophren* OR ((mental* OR developmental* OR intellect* OR cognit* OR psychomotor*) ADJ3 (disease* OR disorder* OR deficien* OR disab* OR impair* OR retard* OR handicap*)) OR psychiatr*).ab,ti.) AND (Ethics/ OR Bioethics/ OR Information Dissemination/ OR Stigma/ OR Privacy/ OR (ethic* OR stigma OR stigmatiz* OR discrimination* OR bioethic* OR disclos* OR (data ADJ3 (shar*)) OR ((incidental OR unsolicit* OR secondar* OR ancillary) ADJ3 finding*) OR privacy OR recontact OR re-contact OR consent OR (return ADJ3 (result* OR finding*)) OR (duty ADJ3 warn) OR (information* ADJ3 disseminat*)).ab,ti.) AND (100.ag. OR 200.ag. OR exp "Parent Child Relations"/ OR exp "Pediatrics"/ OR exp "Child Welfare"/ OR " Childhood Development "/ OR exp "Child Care"/ OR "Childrearing Practices"/ OR "Childrearing Attitudes"/ OR "Child Psychiatry"/ OR "Child Psychology"/ OR "Pediatrics"/ OR exp OR (adolescen* OR infan* OR newborn* OR (new ADJ born*) OR baby OR babies OR neonat* OR child* OR kid OR kids OR toddler* OR teen* OR boy* OR girl* OR minors OR underag* OR (under ADJ1 (age* OR aging)) OR juvenil* OR youth* OR kindergar* OR puber* OR pubescen* OR prepubescen* OR prepubert* OR pediatric* OR paediatric* OR school* OR preschool* OR highschool*).ab,ti.) AND (Genetics/ OR Genome/ OR Genetic Counseling/ OR Genetic Testing/ OR (genetic* OR genomic* OR genome* OR exome OR sequenc* OR (copy ADJ3 number* ADJ3 (variation* OR variant*)) OR (induc* ADJ3 pluripotent* ADJ3 (stem-cell* OR stemcell*)) OR deletion OR duplication OR biobank* OR bio-bank* OR biological sample* OR biomaterial).ab,ti.) AND english.la.

Web of science 550

TS=(((autis* OR schizophren* OR ((mental* OR developmental* OR intellect* OR cognit* OR psychomotor*) NEAR/2 (disease* OR disorder* OR deficien* OR disab* OR impair* OR retard* OR handicap*)) OR psychiatr*)) AND ((ethic* OR stigma OR stigmatiz* OR discrimination* OR bioethic* OR disclos* OR (data NEAR/2 (shar*)) OR ((incidental OR unsolicit* OR secondar* OR ancillary) NEAR/2 finding*) OR privacy OR recontact OR re-contact OR consent OR (return NEAR/2 (result* OR finding*)) OR (duty NEAR/2 warn) OR (information* NEAR/2 disseminat*))) AND ((adolescen* OR infan* OR newborn* OR (new NEAR/1 born*) OR baby OR babies OR neonat* OR child* OR kid OR kids OR toddler* OR teen* OR boy* OR girl* OR minors OR underag* OR (under NEAR/1 (age* OR aging)) OR juvenil* OR youth* OR kindergar* OR puber* OR pubescen* OR prepubescen* OR prepubert* OR pediatric* OR paediatric* OR school* OR preschool* OR highschool*)) AND ((genetic* OR genomic* OR genome* OR exome OR sequenc* OR (copy NEAR/2 number* NEAR/2 (variation* OR variant*)) OR (induc* NEAR/2 pluripotent* NEAR/2 (stem-cell* OR stemcell*)) OR deletion OR duplication OR biobank* OR bio-bank* OR "biological sample*" OR biomaterial)))

Google scholar 200

autism|schizophrenia|"mental|developmental|intellectual|cognitive disease|disorder|deficiency|handicap"|psychiatry ethics|bioethics adolescents|infants|children|pediatric|paediatric genetics|genomics|genome|exome|"copy number variation|variants"
