# Supplementary material for: Ethical issues in genomics research on neurodevelopmental disorders: a critical interpretive review
Source: Hum Genomics. 2021 Mar 12;15:16. doi: 10.1186/s40246-021-00317-4 (PMC7953558; doi:10.1186/s40246-021-00317-4)
Supplement: Supplementary file 2 — Additional file 2. The file includes overview of the search results per database. Overview of the search results. [file 40246_2021_317_MOESM2_ESM.pdf]

Additional file 2: Overview of the search results per database

Databases	# of refs before de-duplication	# of refs after de-duplication
embase.com	675	669
Medline Ovid	691	306
PsycINFO Ovid	338	168
Web of science	550	240
Google scholar	200	191
**Total**	**2454**	**1574**
