# Supplementary material for: Ethical issues in genomics research on neurodevelopmental disorders: a critical interpretive review
Source: Hum Genomics. 2021 Mar 12;15:16. doi: 10.1186/s40246-021-00317-4 (PMC7953558; doi:10.1186/s40246-021-00317-4)
Supplement: Supplementary file 3 — Additional file 3. This file presents a flow diagram of screening process for identified articles. Flow diagram of screening process. [file 40246_2021_317_MOESM3_ESM.pdf]

Additional file 3: Flow diagram of screening process for identified articles

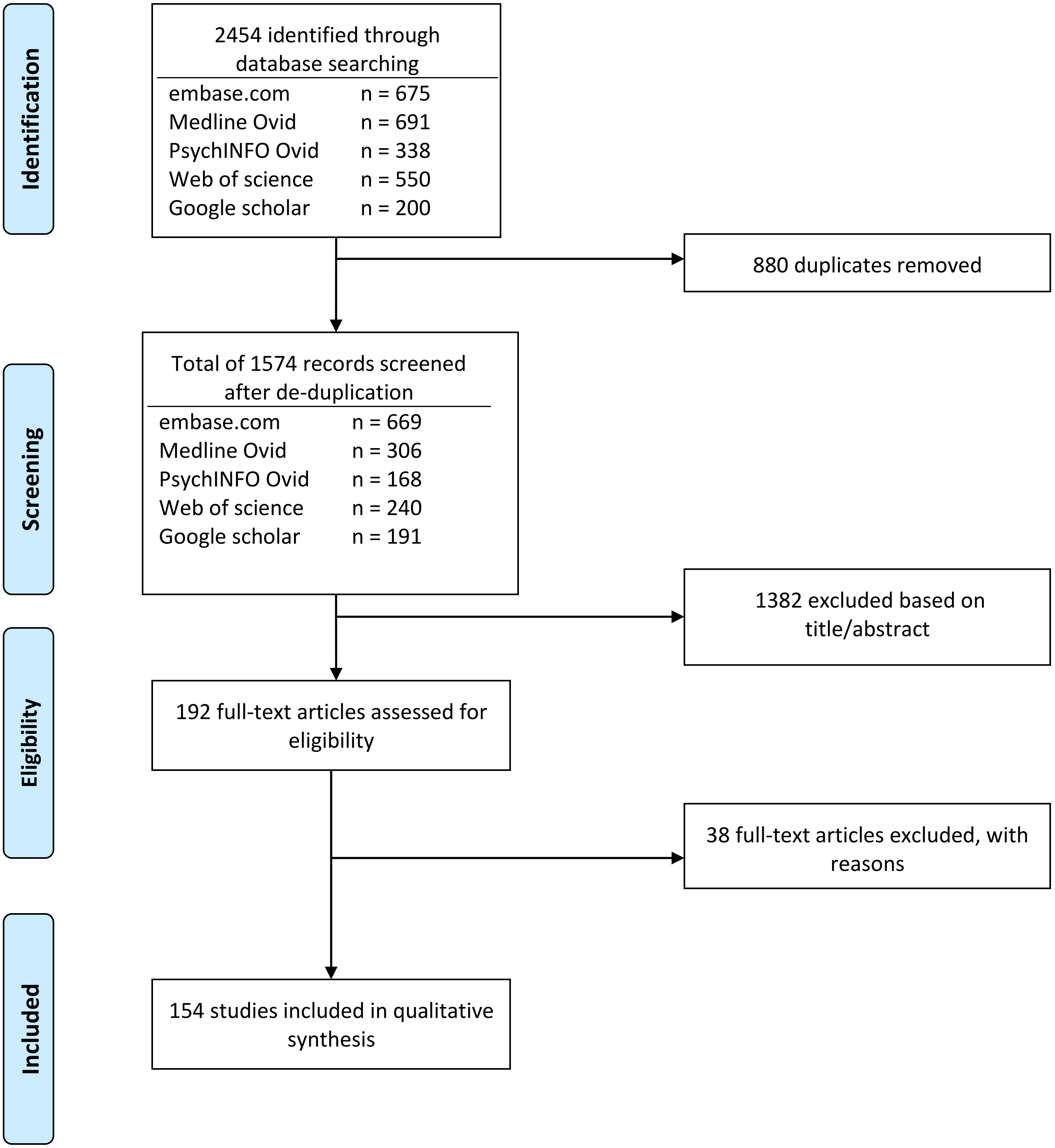

Adapted from: Moher D, Liberati A, Tetzlaff J, Altman DG, The PRISMA Group (2009). Preferred Reporting Items for Systematic Reviews and Meta-Analyses: The PRISMA Statement. PLoS Med 6(7): e1000097. doi:10.1371/journal.pmed1000097

For more information, visit www.prisma-statement.org.
