# Supplementary material for: Ethical issues in genomics research on neurodevelopmental disorders: a critical interpretive review
Source: Hum Genomics. 2021 Mar 12;15:16. doi: 10.1186/s40246-021-00317-4 (PMC7953558; doi:10.1186/s40246-021-00317-4)
Supplement: Supplementary file 4 — Additional file 4. The file includes list of publications included in the analysis. List of publications. [file 40246_2021_317_MOESM4_ESM.pdf]

Additional file 4: List of publications included in the analysis

1. Ahn JW, Bint S, Bergbaum A, Mann K, Hall RP, Ogilvie CM. Array CGH as a first line diagnostic test in place of karyotyping for postnatal referrals - Results from four years' clinical application for over 8,700 patients. Mol Cytogenet. 2013;6(1).
2. Akutagava-Martins GC, Salatino-Oliveira A, Kieling CC, Rohde LA, Hutz MH. Genetics of attention-deficit/hyperactivity disorder: current findings and future directions. Expert Review of Neurotherapeutics. 2013;13(4):435-45.
3. Al Mutairi F, Alfadhel M, Nashabat M, El-Hattab AW, Ben-Omran T, Hertecant J, et al. Phenotypic and Molecular Spectrum of Aicardi-Goutières Syndrome: A Study of 24 Patients. Pediatr Neurol. 2018;78:35-40.
4. Al-jawahiri R, Milne E. Resources available for autism research in the big data era: a systematic review. Peerj. 2017;5.
5. Alpert JE, Biggs MM, Davis L, Shores-Wilson K, Harlan WR, Schneider GW, et al. Enrolling research subjects from clinical practice: ethical and procedural issues in the Sequenced Treatment Alternatives to Relieve Depression (STAR*D) trial. Psychiatry Res. 2006;141(2):193-200.
6. Anders S, Kinney DK. Abnormal immune system development and function in schizophrenia helps reconcile diverse findings and suggests new treatment and prevention strategies Review. Brain Res. 2015;1617:93-112.
7. Anderson IJ, Matteson KJ. New directions in cytogenetic and molecular testing of the neonate. Semin Perinatol. 2005;29(3 SPEC. ISS.):144-9.
8. Appelbaum PS. Ethical issues in psychiatric genetics. J Psychiatr Pract. 2004;10(6):343-51.
9. Arnold LE, Stoff DM, Cook E, Cohen DJ. Ethical issues in biological psychiatric research with children and adolescents. Journal of the American Academy of Child and Adolescent Psychiatry. 1995;34(7):929-39.
10. Asadollahi R, Oneda B, Joset p, Azzarello-Burri S, Bartholdi D, Steindl K, et al. The clinical significance of small copy number variants in neurodevelopmental disorders. J Med Genet. 2014;51(10):677-88.
11. Athanasiu L, Giddaluru S, Fernandes C, Christoforou A, Reinvang I, Lundervold AJ, et al. A genetic association study of CSMD1 and CSMD2 with cognitive function. Brain Behav Immun. 2017;61:209-16.
12. Au PYB, You J, Caluseriu O, Schwartzentruber J, Majewski J, Bernier FP, et al. GeneMatcher aids in the identification of a new malformation syndrome with intellectual disability, unique facial dysmorphisms, and skeletal and connective tissue abnormalities caused by de novo variants in HNRNPK. Hum Mutat. 2015;36(10):1009-14.
13. Austin J, Inglis A, Hadjipavlou G. Genetic counseling for common psychiatric disorders: an opportunity for interdisciplinary collaboration. Am J Psychiatry. 2014;171(5):584-5.
14. Babameto-Laku A, Grabova S, Vyshka G, Kruja J. Epileptic Seizures Associated with Chromosomal Abnormalities Detected by Array Comparative Genomic Hybridization in Five Albanian Children. Journal of Pediatric Epilepsy. 2017;6(3):156-60.
15. Bacchelli C, Williams HJ. Opportunities and technical challenges in next-generation sequencing for diagnosis of rare pediatric diseases. Expert Review of Molecular Diagnostics. 2016;16(10):1073-82.
16. Baret L, Godard B. Opinions and intentions of parents of an autistic child toward genetic research results: Two typical profiles. Eur J Hum Genet. 2011;19(11):1127-32.
17. Bassett AS, Chow EWC, Weksberg R, Brzustowicz L. Unravelling tihe mysteries of schizophrenia: Advances from genetic studies. International Seminar on Nuclear War and Planetary Emergencies - 27th Session. Science and Culture Series: Nuclear Strategy and Peace Technology. Singapore: World Scientific Publ Co Pte Ltd; 2003. p. 471-83.
18. Bauer SC, Msall ME. Genetic testing for autism spectrum disorders. Dev Disabil Res Rev. 2011;17(1):3-8.
19. Baughman ST, Morris E, Jensen K, Austin J. Disclosure of psychiatric manifestations of 22q11.2 deletion syndrome in medical genetics: A 12-year retrospective chart review. Am J Med Genet Part A. 2015;167(10):2350-6.
20. Beale S, Sanderson D, Sanniti A, Dundar Y, Boland A. A scoping study to explore the cost-effectiveness of next-generation sequencing compared with traditional genetic testing for the diagnosis of learning disabilities in children. Health Technol Assess. 2015;19(46).
21. Belmonte MK, Mazziotta JC, Minshew NJ, Evans AC, Courchesne E, Dager SR, et al. Offering to share: how to put heads together in autism neuroimaging. J Autism Dev Disord. 2008;38(1):2-13.
22. Ben Said M, Robel L, Golse B, Jais JP. Strengthening Data Confidentiality and Integrity Protection in the Context of a Multi-Centric Information System Dedicated to Autism Spectrum Disorder. Stud Health Technol Inform. 2017;245:1133-7.
23. Bennett L, Thirlaway K, Murray AJ. The stigmatising implications of presenting schizophrenia as a genetic

disease. J Genet Couns. 2008;17(6):550-9.
24. Berryessa CM, Cho MK. Ethical, legal, social, and policy implications of behavioral genetics. Annu Rev Genomics Hum Genet2013. p. 515-34.
25. Beunders G, Dekker M, Haver O, Meijers-Heijboer HJ, Henneman L. Recontacting in light of new genetic diagnostic techniques for patients with intellectual disability: Feasibility and parental perspectives. European Journal of Medical Genetics. 2018;61(4):213-8.
26. Biesecker BB, Peay HL. Ethical issues in psychiatric genetics research: points to consider. Psychopharmacology. 2003;171(1):27-35.
27. Botkin JR, Belmont JW, Berg JS, Berkman BE, Bombard Y, Holm IA, et al. Points to Consider: Ethical, Legal, and Psychosocial Implications of Genetic Testing in Children and Adolescents. American Journal of Human Genetics. 2015;97(1):6-21.
28. Bourgeron T. Current knowledge on the genetics of autism and propositions for future research Review. C R Biol. 2016;339(7-8):300-7.
29. Bowdin S, Ray PN, Cohn RD, Meyn MS. The Genome Clinic: A Multidisciplinary Approach to Assessing the Opportunities and Challenges of Integrating Genomic Analysis into Clinical Care. Human Mutation. 2014;35(5):513-9.
30. Brothers KB, East KM, Kelley WV, Wright MF, Westbrook MJ, Rich CA, et al. Eliciting preferences on secondary findings: The Preferences Instrument for Genomic Secondary Results. Gen Med. 2017;19(3):337-44.
31. Bruno DL, White SM, Ganesamoorthy D, Burgess T, Butler K, Corrie S, et al. Pathogenic aberrations revealed exclusively by single nucleotide polymorphism (SNP) genotyping data in 5000 samples tested by molecular karyotyping. J Med Genet. 2011;48(12):831-9.
32. Castro-Gago M. Neurología pediátrica y genética: introducción. Revista De Neurologia. 2002;35(2):145-8.
33. Chen DT, Miller FG, Rosenstein DL. Ethical aspects of research into the etiology of autism Review. Ment Retard Dev Disabil Res Rev. 2003;9(1):48-53.
34. Chen LS, Xu L, Huang TY, Dhar SU. Autism genetic testing: a qualitative study of awareness, attitudes, and experiences among parents of children with autism spectrum disorders. Genetics in Medicine. 2013.
35. Christenhusz GM, Devriendt K, Peeters H, Van Esch H, Dierickx K. The communication of secondary variants: Interviews with parents whose children have undergone array-CGH testing. Clin Genet. 2014;86(3):207-16.
36. Christenhusz GM, Devriendt K, Van Esch H, Dierickx K. Focus group discussions on secondary variants and next-generation sequencing technologies. Eur J Med Genet. 2015;58(4):249-57.
37. Chung BHY, Tao VQ, Tso WWY. Copy number variation and autism: New insights and clinical implications. J Formos Med Assoc. 2014;113(7):400-8.
38. Clarke AJ. Managing the ethical challenges of next-generation sequencing in genomic medicine. British Medical Bulletin. 2014;111(1):17-30.
39. Coors ME, Raymond KM. Substance use disorder genetic research: Investigators and participants grapple with the ethical issues. Psychiatr Genet. 2009;19(2):83-90.
40. Corcoran C, Malaspina D, Hercher L. Prodromal interventions for schizophrenia vulnerability: The risks of being "at risk". Schizophr Res. 2005;73(2-3):173-84.
41. Cornelis C, Tibben A, Dondorp W, Van Haelst M, Bredenoord AL, Knoers N, et al. Whole-exome sequencing in pediatrics: Parents' considerations toward return of unsolicited findings for their child. Eur J Hum Genet. 2016;24(12):1681-7.
42. Cunningham JL, Zanzi M, Willebrand M, Ekselius L, Ramklint M. No regrets: Young adult patients in psychiatry report positive reactions to biobank participation. Erratum appears in BMC Psychiatry. 2017 Feb 20;17 (1):74; PMID: 28219362. BMC Psychiatry. 2017;17(1):21.
43. Curran WJ. Ethical and legal considerations in high risk studies of schizophrenia. Schizophr Bull. 1974(10):74-92.
44. Daley TC, Singhal N, Krishnamurthy V. Ethical considerations in conducting research on autism spectrum disorders in low and middle income countries. J Autism Dev Disord. 2013;43(9):2002-14.
45. Drury S, Cuthbert B. Advancing pediatric psychiatry research: Linking neurobiological processes to novel treatment and diagnosis through the Research Domain Criteria (RDoC) project. Ther Innov Regul Sci. 2015;49(5):643-6.
46. Dunn LB, Candilis PJ, Roberts LW. Emerging empirical evidence on the ethics of schizophrenia research: academic.oup.com; 2005.
47. Faux D, Schoch K, Eubanks S, Hooper SR, Shashi V. Assessment of parental disclosure of a 22q11.2 deletion syndrome diagnosis and implications for clinicians. J Genet Couns. 2012;21(6):835-44.
48. Firth HV, Richards SM, Bevan AP, Clayton S, Corpas M, Rajan D, et al. DECIPHER: Database of Chromosomal

Imbalance and Phenotype in Humans Using Ensembl Resources. Am J Hum Genet. 2009;84(4):524-33.
49. Fischbach GD, Lord C. The Simons Simplex Collection: a resource for identification of autism genetic risk factors. Neuron. 2010;68(2):192-5.
50. Fischbach RL, Harris MJ, Ballan MS, Fischbach GD, Link BG. Is there concordance in attitudes and beliefs between parents and scientists about autism spectrum disorder? Autism. 2016;20(3):353-63.
51. Foo JN, Liu JJ, Tan EK. Next-generation sequencing diagnostics for neurological diseases/disorders: from a clinical perspective. Human Genetics. 2013;132(7):721-34.
52. Fuentes J, Martin-Arribas MC. Bioethical issues in neuropsychiatric genetic disorders. Child and Adolescent Psychiatric Clinics of North America. 2007;16(3):649-+.
53. Gershon ES, Alliey-Rodriguez N. New ethical issues for genetic counseling in common mental disorders Review. Am J Psychiatry. 2013;170(9):968-76.
54. Giarelli E, Reiff M. Mothers' appreciation of chromosomal microarray analysis for autism spectrum disorder. Journal for Specialists in Pediatric Nursing. 2015;20(4):244-58.
55. Godfrey E, Clark P. Developing standards for chromosomal microarray testing counselling in paediatrics. Acta Paediatr Int J Paediatr. 2014;103(6):574-7.
56. Goodwin J, McCormack L, Campbell LE. "You Don't Know Until You Get There": The Positive and Negative "Lived" Experience of Parenting an Adult Child With 22q11.2 Deletion Syndrome. Health Psychology. 2017;36(1):45-54.
57. Goodwin J, Schoch K, Shashi V, Hooper SR, Morad O, Zalevsky M, et al. A tale worth telling: The impact of the diagnosis experience on disclosure of genetic disorders. J Intellect Disabil Res. 2015;59(5):474-86.
58. Green RC, Berg JS, Berry GT, Biesecker LG, Dimmock DP, Evans JP, et al. Exploring concordance and discordance for return of incidental findings from clinical sequencing. Gen Med. 2012;14(4):405-10.
59. Green RC, Berg JS, Grody WW, Kalia SS, Korf BR. ACMG recommendations for reporting of incidental findings in clinical exome and genome sequencing. Genetics in Medicine. 2013;15(7):565–74.
60. Grisart B, Willatt L, Destrée A, Fryns JP, Rack K, De Ravel T, et al. 17q21.31 microduplication patients are characterised by behavioural problems and poor social interaction. J Med Genet. 2009;46(8):524-30.
61. H. KSY. Evidence-based ethics for neurology and psychiatry research. NeuroRx: The Journal of the American Society for Experimental NeuroTherapeutics. 2004;1(3):372–7.
62. Haas OA, Bodamer O. Genetics in pediatrics as an interaction between clinic and laboratory. Monatsschrift Kinderheilkunde. 2008;156(4):323-+.
63. Harris ED, Ziniel SI, Amatruda JG, Clinton CM, Savage SK, Taylor PL, et al. The beliefs, motivations, and expectations of parents who have enrolled their children in a genetic biorepository. Gen Med. 2012;14(3):330-7.
64. Hart SJ, Schoch K, Shashi V, Callanan N. Communication of Psychiatric Risk in 22q11.2 Deletion Syndrome: A Pilot Project. J Genet Couns. 2016;25(1):6-17.
65. Hayeems RZ, Miller FA, Li L, Bytautas JP. Not so simple: a quasi-experimental study of how researchers adjudicate genetic research results. Eur J Hum Genet. 2011;19(7):740-7.
66. Hens K, Peeters H, Dierickx K. The ethics of complexity. Genetics and autism, a literature review. Am J Med Genet Part B Neuropsychiatr Genet. 2016;171(3):305-16.
67. Hens K, Peeters H, Dierickx K. Genetic testing and counseling in the case of an autism diagnosis: A caregivers perspective. Eur J Med Genet. 2016;59(9):452-8.
68. Hens K, Peeters H, Dierickx K. Shooting a moving target. Researching autism genes: An interview study with professionals. Eur J Med Genet. 2016;59(1):32-8.
69. Hercher L, Bruenner G. Living with a child at risk for psychotic illness: The experience of parents coping with 22q11 deletion syndrome: An exploratory study. American Journal of Medical Genetics Part A. 2008;146A(18):2355-60.
70. Hinton VJ. Ethics of neuroimaging in pediatric development. Brain and Cognition. 2002;50:455–68.
71. Hoge SK, Appelbaum PS. Ethical, legal, and social implications of psychiatric genetics and genetic counseling. In: Smoller J TM, and Rosen-Sheidley B editor. Psychiatric Genetics: Applications in Clinical Practic2008. p. 255-76.
72. Hoge SK, Appelbaum PS. Ethics and neuropsychiatric genetics: A review of major issues. Int J Neuropsychopharmacol. 2012;15(10):1547-57.
73. Holland A, Clare ICH. The Human Genome Project: considerations for people with intellectual. Journal of Intellectual Disability Research. 2003;47:515-25.
74. Holm IA, Iles BR, Ziniel SI, Bacon PL, Savage SK, Christensen KD, et al. Participant Satisfaction With a Preference-Setting Tool for the Return of Individual Research Results in Pediatric Genomic Research. Journal of Empirical Research on Human Research Ethics. 2015;10(4):414-26.
75. Houdayer F, Gargiulo M, Frischmann M, Labalme A, Decullier E, Cordier MP, et al. The psychological impact

of cryptic chromosomal abnormalities diagnosis announcement. European Journal of Medical Genetics. 2013;56(11):585-90.
76. Jensen PS, Mrazek DA. Research and Clinical Perspectives in Defining and Assessing Mental Disorders in Children and Adolescents. Toward a new diagnostic system for child psychopathology: Moving beyond the DSM: Guilford Press; 2006. p. 11-37.
77. Johannessen J, Nærland T, Bloss C, Rietschel M, Strohmaier J, Gjevik E, et al. Parents' attitudes toward genetic research in autism spectrum disorder. Psychiatr Genet. 2016;26(2):74-80.
78. Johannessen J, Naerland T, Hope S, Torske T, Hoyland AL, Strohmaier J, et al. Parents' Attitudes toward Clinical Genetic Testing for Autism Spectrum Disorder-Data from a Norwegian Sample. International Journal of Molecular Sciences. 2017;18(5).
79. Jordan BR, Tsai DF. Whole-genome association studies for multigenic diseases: ethical dilemmas arising from commercialization--the case of genetic testing for autism. J Med Ethics. 2010;36(7):440-4.
80. Kaufman D, Geller G, Leroy L, Murphy J, Scott J, Hudson K. Ethical implications of including children in a large biobank for genetic-epidemiologic research: A qualitative study of public opinion. Am J Med Genet Part C Semin Med Genet. 2008;148(1):31-9.
81. L. Z, A. T, W. S, G. B. Studying the emergence of autism spectrum disorders in high-risk infants: methodological and practical issues. Journal of Autism and Developmental Disorders. 2007;37:466–80.
82. Laegsgaard MM, Kristensen AS, Mors O. Potential Consumers' Attitudes Toward Psychiatric Genetic Research and Testing and Factors Influencing Their Intentions to Test. Genetic Testing and Molecular Biomarkers. 2009;13(1):57-65.
83. Laegsgaard MM, Mors O. Psychiatric genetic testing: Attitudes and intentions among future users and providers. Am J Med Genet Part B Neuropsychiatr Genet. 2008;147(3):375-84.
84. Lajonchere CM, Consortium A. Changing the landscape of autism research: the autism genetic resource exchange. Neuron. 2010;68(2):187-91.
85. Lawrence RE, Appelbaum PS. Genetic testing in psychiatry: A review of attitudes and beliefs. Psychiatry (USA). 2011;74(4):315-31.
86. Levenseller BL, Soucier DJ, Miller VA, Harris D. Stakeholders' opinions on the implementation of pediatric whole exome sequencing: implications for informed consent. Journal of genetic counseling 2014;23:552–65.
87. Liu EY, Scott CT. Great expectations: autism spectrum disorder and induced pluripotent stem cell technologies Review. Stem cell rev. 2014;10(2):145-50.
88. Lloyd K, McGregor J, John A, Craddock N, Walters JT, Linden D, et al. A national population-based e-cohort of people with psychosis (PsyCymru) linking prospectively ascertained phenotypically rich and genetic data to routinely collected records: Overview, recruitment and linkage. Schizophr Res. 2015;166(1-3):131-6.
89. Longstaff H, Khramova V, Portales-Casamar E, Illes J. Sharing with more caring: Coordinating and improving the ethical governance of data and biomaterials obtained from children. PLoS ONE. 2015;10(7).
90. Loth E, Murphy DG, Spooren W. Defining Precision Medicine Approaches to Autism spectrum Disorders: concepts and challenges. Frontiers in Psychiatry. 2016;7.
91. Manzini A, Vears DF. Predictive Psychiatric Genetic Testing in Minors: An Exploration of the Non-Medical Benefits. Erratum appears in J Bioeth Inq. 2018 Mar 21;:; PMID: 29564710. J Bioeth Inq. 2018;15(1):111-20.
92. Martin N, Mikhaelian M, Cytrynbaum C, Shuman C, Chitayat DA, Weksberg R, et al. 22q11.2 deletion syndrome: attitudes towards disclosing the risk of psychiatric illness. J Genet Couns. 2012;21(6):825-34.
93. McCabe LL, McCabe ERB. Down syndrome: Issues to consider in a national registry, research database and biobank. Mol Genet Metab. 2011;104(1-2):10-2.
94. McGuire AL, Oliver JM, Slashinski MJ, Graves JL, Wang T, Kelly PA, et al. To share or not to share: a randomized trial of consent for data sharing in genome research. Genet Med. 2011;13(11):948-55.
95. McMahon WM, Baty BJ, Botkin J. Genetic counseling and ethical issues for autism Review. Am J Med Genet C Semin Med Genet. 2006;142C(1):52-7.
96. Miller FA, Hayeems RZ, Bytautas JP. What is a meaningful result Disclosing the results of genomic research in autism to research participants. Eur J Hum Genet. 2010;18(8):867-71.
97. Miller FA, Hayeems RZ, Li L, Bytautas JP. What does 'respect for persons' require? Attitudes and reported practices of genetics researchers in informing research participants about research. Journal of Medical Ethics. 2011.
98. Morris E, Inglis A, Friedman J, Austin J. Discussing the psychiatric manifestations of 22q11.2 deletion syndrome: An exploration of clinical practice among medical geneticists. Gen Med. 2013;15(9):713-20.
99. Nguyen K, Putoux A, Busa T, Cordier MP, Sigaudy S, Till M, et al. Incidental findings on array comparative

genomic hybridization: Detection of carrier females of dystrophinopathy without any family history. Clin Genet. 2015;87(5):488-91.
100. Novikova SI, Richman DM, Supekar K, Barnard-Brak L, Hall D. NDAR: A Model Federal System for Secondary Analysis in Developmental Disabilities Research. International Review of Research in Developmental Disabilities. 2013;45:123-53.
101. O'Lonergan TA, Milgrom H. Ethical considerations in research involving children. Curr Allergy Asthma Rep. 2005;5(6):451-8.
102. Pagnamenta AT, Holt R, Yusuf M, Pinto D, Wing K, Betancur C, et al. A family with autism and rare copy number variants disrupting the Duchenne/Becker muscular dystrophy gene DMD and TRPM3. J Neurodev Disord. 2011;3(2):124-31.
103. Patenaude AF. Pediatric psychology training and genetics: What will twenty-first-century pediatric psychologists need to know? J Pediatr Psychol. 2003;28(2):135-45.
104. Payakachat N, Tilford JM, Ungar WJ. National Database for Autism Research (NDAR): Big Data Opportunities for Health Services Research and Health Technology Assessment. Pharmacoeconomics. 2016;34(2):127-38.
105. Pellicano E, Stears M. Bridging autism, science and society: moving toward an ethically informed approach to autism research Review. Autism Res. 2011;4(4):271-82.
106. Pendergrass S, Girirajan S, Selleck S. Uncovering the etiology of autism spectrum disorders: genomics, bioinformatics, environment, data collection and exploration, and future possibilities. Pac Symp Biocomput. 2014:422-6.
107. Perry A. Autism beyond pediatrics: Why bioethicists ought to rethink consent in light of chronicity and genetic identity. Bioethics. 2012;26(5):236-41.
108. Petrin AL, Daack-Hirsch S, L'Heureux J, Murray JC. A case of 3q29 microdeletion syndrome involving oral cleft inherited from a nonaffected mosaic parent: molecular analysis and ethical implications. Cleft Palate Craniofac J. 2011;48(2):222-30.
109. Plomin R, Davis OSP. The future of genetics in psychology and psychiatry: microarrays, genome-wide association, and non-coding RNA. ... of Child Psychology and Psychiatry. 2009.
110. Post SG. Preventing schizophrenia and Alzheimer disease: comparative ethics. Schizophr Res. 2001;51(1):103-8.
111. Press KR, Wieczorek L, Hoover-Fong J, Bodurtha J, Taylor L. Overview: Referrals for genetic evaluation from child psychiatrists. Child Adolesc Psychiatry Ment Health. 2016;10(1).
112. Rapp R. Big data, small kids: Medico-scientific, familial and advocacy visions of human brains. Biosocieties. 2016;11(3):296-316.
113. Reiff M, Bugos E, Giarelli E, Bernhardt BA, Spinner NB, Sankar PL, et al. "Set in Stone" or "Ray of Hope": Parents' Beliefs About Cause and Prognosis After Genomic Testing of Children Diagnosed with ASD. Journal of Autism and Developmental Disorders. 2017;47(5):1453-63.
114. Ries NM, LeGrandeur J, Caulfield T. Handling ethical, legal and social issues in birth cohort studies involving genetic research: responses from studies in six countries. BMC Med Ethics. 2010;11(1):4.
115. Roberts JS, Uhlmann WR. Genetic susceptibility testing for neurodegenerative diseases: Ethical and practice issues. Prog Neurobiol. 2013;110:89-101.
116. Roberts LW. Ethics and mental illness research. Psychiatr Clin North Am. 2002;25(3):525-45.
117. Rossi J, Newschaffer C, Yudell M. Autism spectrum disorders, risk communication, and the problem of inadvertent harm. Kennedy Inst Ethics J. 2013;23(2):105-38.
118. Rothenberger LG. Molecular Genetics Research in ADHD: Ethical Considerations Concerning Patients' Benefit and Resource Allocation. American Journal of Medical Genetics Part B-Neuropsychiatric Genetics. 2012;159B(8):885-95.
119. Sampieri K, Meloni I, Scala E, Ariani F, Caselli R, Pescucci C, et al. Italian Rett database and biobank. Human Mutation. 2007;28(4):329-35.
120. Sapp JC, Dong D, Stark C, Ivey LE, Hooker G. Parental attitudes, values, and beliefs toward the return of results from exome sequencing in children. ... genetics. 2014.
121. Satterthwaite TD, Connolly JJ, Ruparel K, Calkins ME, Jackson C, Elliott MA, et al. The Philadelphia Neurodevelopmental Cohort: A publicly available resource for the study of normal and abnormal brain development in youth. Neuroimage. 2016;124(Pt B):1115-9.
122. Schachar R, Soreni N. Next generation sequencing and the child and youth psychiatrist. Journal of the Canadian Academy of Child and Adolescent Psychiatry / Journal de l'Academie canadienne de psychiatrie de l'enfant

et de l'adolescent. 2015;24(2):83.
123. Scherer SW, Dawson G. Risk factors for autism: translating genomic discoveries into diagnostics Review. Hum Genet. 2011;130(1):123-48.
124. Schiavone S, Neri M, Pomara C, Riezzo I, Trabace L, Turillazzi E. Personalized medicine in the paediatric population: The balance between pharmacogenetic progress and bioethics. Curr Pharm Biotechnol. 2017;18(3):253-62.
125. Sexton AC, Metcalfe SA. Disclosing genetic research results after death of pediatric patients. J Am Med Assoc. 2008;300(14):1693-5.
126. Sherr EH, Michelson DJ, Shevell MI, Moeschler JB, Gropman AL, Ashwal S. Neurodevelopmental disorders and genetic testing: current approaches and future advances Review. Ann Neurol. 2013;74(2):164-70.
127. Simons Vip C. Simons Variation in Individuals Project (Simons VIP): a genetics-first approach to studying autism spectrum and related neurodevelopmental disorders. Neuron. 2012;73(6):1063-7.
128. Sondheimer AN, Klykylo WM. The ethics committees of the American Academy of Child and Adolescent Psychiatry and the American Psychiatric Association: History, process, education, and advocacy. Child and Adolescent Psychiatric Clinics of North America. 2008;17(1):225-+.
129. State MW, Lombroso PJ, Pauls DL, Leckman JF. The genetics of childhood psychiatric disorders: A decade of progress. J Am Acad Child Adolesc Psychiatry. 2000;39(8):946-62.
130. Statham H, Ponder M, Richards M, Hallowell N, Raymond FL. A family perspective of the value of a diagnosis for intellectual disability: experiences from a genetic research study. British Journal of Learning Disabilities. 2011;39(1):46-56.
131. Stuckey H, Williams JL, Fan AL, Rahm AK, Green J, Feldman L, et al. Enhancing genomic laboratory reports from the patients' view: A qualitative analysis. Am J Med Genet A. 2015;167A(10):2238-43.
132. Sundby A, Boolsen MW, Burgdorf KS, Ullum H, Hansen TF, Mors O. Attitudes of stakeholders in psychiatry towards the inclusion of children in genomic research. Hum Genomics. 2018;12(1):12.
133. Tabor HK, Brazg T, Crouch J, Namey EE, Fullerton SM, Beskow LM, et al. Parent perspectives on pediatric genetic research and implications for genotype-driven research recruitment. J Empir Res Hum Res Ethics. 2011;6(4):41-52.
134. Tabor HK, Cho MK. Ethical implications of array comparative genomic hybridization in complex phenotypes: points to consider in research Review. Genet Med. 2007;9(9):626-31.
135. Tervo RC, Wojda P. The principle of double effect, genetic testing, and global developmental delay. J Child Neurol. 2009;24(8):1030-6.
136. Tervo RC, Wojda P. Genetic testing in global developmental delay: the well-intended clinician and the principle of double effect. Acta Paediatr. 2009;98(8):1251-3.
137. Thomas CR. Epigenetics and Child Psychiatry: Ethical and Legal Issues. Behav Sci Law. 2015;33(5):644-52.
138. Uhlmann WR, Roberts JS. Ethical issues in neurogenetics. Handb clin neurol. 2018;147:23-36.
139. Ungar WJ. Next Generation Sequencing and Health Technology Assessment in Autism Spectrum Disorder. Journal of the Canadian Academy of Child and Adolescent Psychiatry. 2015;24(2):123-7.
140. United Kingdom. Nuffield Council on B. Mental disorders and genetics: the ethical context: conclusions and recommendations. J Int Bioethique. 2001;12(2):89-92.
141. Valente EM, Ferraris A, Dallapiccola B. Genetic testing for paediatric neurological disorders. Lancet Neurol. 2008;7(12):1113-26.
142. Veltman JA, Cuppen E, Vrijenhoek T. Challenges for implementing next-generation sequencing-based genome diagnostics: it's also the people, not just the machines. Personalized Medicine. 2013;10(5):473-84.
143. Vissers L, van Nimwegen KJM, Schieving JH, Kamsteeg EJ, Kleefstra T, Yntema HG, et al. A clinical utility study of exome sequencing versus conventional genetic testing in pediatric neurology. Genetics in Medicine. 2017;19(9):1055-63.
144. Voineagu I, Yoo HJ. Current progress and challenges in the search for autism biomarkers Review. Dis Markers. 2013;35(1):55-65.
145. Walsh P, Elsabbagh M, Bolton P, Singh I. In search of biomarkers for autism: scientific, social and ethical challenges. Nature Reviews …. 2011.
146. Wilfond B, Ross LF. From genetics to genomics: ethics, policy, and parental decision-making. Journal of Pediatric Psychology. 2008;34(6):639-47.
147. Wright CF, Fitzgerald TW, Jones WD, Clayton S, McRae JF, Van Kogelenberg M, et al. Genetic diagnosis of developmental disorders in the DDD study: A scalable analysis of genome-wide research data. Lancet. 2015;385(9975):1305-14.

148. Wright CF, FitzPatrick DR, Firth HV. Paediatric genomics: diagnosing rare disease in children. Nature Reviews Genetics. 2018;19(5):253-68.
149. Wright CF, McRae JF, Clayton S, Gallone G, Aitken S, FitzGerald TW, et al. Making new genetic diagnoses with old data: iterative reanalysis and reporting from genome-wide data in 1,133 families with developmental disorders. Genetics in Medicine. 2018.
150. Xiao B, Qiu W, Ji X, Liu X, Huang Z, Liu H, et al. Marked yield of re-evaluating phenotype and exome/target sequencing data in 33 individuals with intellectual disabilities. Am J Med Genet Part A. 2018;176(1):107-15.
151. Yan EG, Munir KM. Regulatory and ethical principles in research involving children and individuals with developmental disabilities. Ethics & Behavior. 2004;14(1):31–49.
152. Yoshida A, Dowa Y, Murakami H, Kosugi S. Obtaining subjects' consent to publish identifying personal information: current practices and identifying potential issues. BMC Med Ethics. 2013;14:47.
153. Yudell M, Tabor HK, Dawson G, Rossi J, Newschaffer C, Working Group in Autism Risk C, et al. Priorities for autism spectrum disorder risk communication and ethics. Autism. 2013;17(6):701-22.
154. Yusuf A, Elsabbagh M. At the cross-roads of participatory research and biomarker discovery in autism: the need for empirical data. Bmc Medical Ethics. 2015;16.
